# Management of ankylosed teeth using the decoronation technique: integrative literature review and case report

**DOI:** 10.1590/2177-6709.28.4.e23spe4

**Published:** 2023-10-09

**Authors:** Eustáquio Afonso ARAÚJO, Gabriel Ferreira Pessoa Carvalho MIRANDA

**Affiliations:** 1 Emeritus professor, Department of Orthodontics, Saint Louis University (St. Louis/MO, USA). Adjunct professor, Department of Orthodontics, University of Pittsburgh (Pittsburgh/PA, USA). Professor, Department of Orthodontics, Faculdade Ciências Médicas de Minas Gerais (Belo Horizonte/MG, Brazil).; University of Pittsburgh, Department of Orthodontics, University of Pittsburgh, Pittsburgh, PA, USA; Department of Orthodontics, Faculdade Ciências Médicas de Minas Gerais, Belo Horizonte, MG, Brazil; 2 Research Scholar, Department of Orthodontics, Saint Louis University (St. Louis/MO, USA).

**Keywords:** Malocclusion, Angle Class II, Tooth ankylosis, Decoronation

## Abstract

**Introduction:**

The decoronation technique has been described in literature since 1984 and, based on the available results, it can lead to considerable benefits for the repair and rehabilitation of ankylosed teeth. Based on these reports, one could expect that this procedure would be well known by the dental community. However, this fact does not seem to be true, and this procedure is not widely used.

**Methods:**

The objective of this paper is to present appropriate literature that discusses decoronation and evaluate the perspectives of the procedure, both in relation to the technique and the long-term benefits for the patient. An integrative literature review at PubMed, ScieELO, and Lilacs databases was performed using the keywords “decoronation”, “ridge preservation decoronation”, “decoronation ankylosis”. In addition, a case report will be presented to demonstrate the technique in a systematic and detailed manner.

**Results:**

Considering the inclusion criteria, 27 articles that present consistency regarding decoronation were selected.

**Conclusion:**

There is scarce availability of scientific works related to the topic, to corroborate and discuss the technique. The present paper reinforces the benefits of this procedure, and revisit decoronation, attempting to provide a possible treatment for ankylosed teeth in growing patients.

## RESUMO

**Introdução:**

A técnica de decoronação é descrita na literatura desde 1984 e, com base nos resultados disponíveis, pode trazer benefícios consideráveis para o reparo e reabilitação de dentes anquilosados. Com base nesses relatos, seria esperado que esse procedimento fosse bem conhecido pela comunidade odontológica. No entanto, isto não parece ser verdadeiro e esse procedimento não é amplamente utilizado.

**Objetivo:**

O objetivo deste artigo é apresentar literatura adequada que discuta a decoronação e avalie as perspectivas do procedimento, tanto em relação à técnica quanto aos benefícios em longo prazo para o paciente.

**Métodos:**

Foi realizada revisão integrativa da literatura nas bases de dados PubMed, SciELO e Lilacs, utilizando as seguintes palavras-chave: “*decoronation*”, “*ridge preservation decoronation*”, “*decoronation ankylosis”*. Além disso, um relato de caso demonstrará a técnica de maneira sistemática e detalhada.

**Resultados:**

Considerando os critérios de inclusão, foram selecionados 27 artigos que apresentam consistência quanto à decoronação.

**Conclusão:**

Há escassa disponibilidade de trabalhos científicos relacionados ao tema para corroborar e discutir a técnica. Esse artigo reforça os benefícios desse procedimento e revisa a decoronação na tentativa de fornecer um possível tratamento para dentes anquilosados em pacientes em crescimento.

## INTRODUCTION

Dental trauma and its consequences are well-described topics in the literature. Among possible complications, ankylosis imposes itself as one of the most severe forms of trauma unfolding. However, little attention has been pointed out on the handling of this problem, especially in children and adolescents in the early growth and development stages. Currently, the available techniques do not demonstrate an acceptable level of predictability, and can lead to negative effects that often outweigh the positive ones.^1^ ^,^ ^2^

Unlike traditional techniques, such as surgical repositioning and bone distraction, decoronation -consisting of crown removal for bone preservation in height and width, maintaining periodontal integrity by inducing bone formation and facilitating the rehabilitation phase- has been established as an alternative that aims to provide benefits for the patient affected by a traumatic injury.^2^ ^,^ ^3^ ^,^ ^4^ However, little has been discussed on this topic, and it is believed that a small portion of orthodontists is aware of this technique.^5^

Thus, the present article aimed to review the literature, presenting and discussing relevant information for the orthodontist, based on credible scientific pieces of evidence that would allow a secure application in private practice. This publication may work as a guide for the orthodontist not only to be familiar with the procedure, but also to be confident when indicating it.

Based on these facts, this article performs an integrative review of the literature on decoronation, including a detailed description of a case report. A thorough overview of what has been published on this topic, how to place it into clinical practice, as well as the benefits of implementing such a technique for patients with ankylosed teeth will be described.

## MATERIALS AND METHODS

The investigation on decoronation-related articles was carried out by accessing the following health databases: PubMed, SciELO, and Lilacs. The specific keywords were “decoronation”, “ridge preservation decoronation”, “decoronation ankylosis”.

To be included in this study, articles should have full text available, be in the English language, and dated from 1984 (the first article published on the topic) to the present date. 

The analysis and synthesis of the data is presented in a descriptive way, making it possible to classify the information, in order to assemble the knowledge produced about the technique of decoronation over the years. 

The variables of interest address: the description of the technique, its indications, the ideal moment to intervene, and the benefits achieved by means of decoronation. 

As a way of illustrating the technique, a clinical case previously treated with decoronation will be described.

## RESULTS

The search for decoronation in the databases found 558 titles: 556 in PubMed, 1 in SciELO and 3 in Lilacs. Of those, one was present in all three databases. After reviewing titles, abstracts and reading the full texts, only 27 fully addressed the inclusion criteria of the present study.

## LITERATURE REVIEW/DISCUSSION

Ankylosis is a relevant aspect of traumatic injuries, and can be stated as a public health issue.^4^ ^,^ ^6^ A 1995 publication demonstrated the worldwide concerns involving the prevalence of ankylosis among children and teenagers after avulsion and reinplantation of permanent teeth.^7^ Recent literature agrees and relate an increase in ankylosis incidence on epidemiological surveys in the current decade.^8^ ^,^ ^9^

Once confirmed its diagnosis, limited alternatives for treatment are available. Among them, surgical removal is the most widespread among dentists, even though it can create negative effects, such as alveolar bone volume loss (height and width)^4^ ^,^ ^10^ that hinders the future rehabilitation process.^2^ ^,^ ^10^ In spite of that, little attention has been paid^4^ ^,^ ^5^ to a conservative and simple technique for the treatment of ankylosed teeth: the so-called decoronation.^2^ ^,^ ^4^ ^,^ ^11^

The decoronation technique was first described in 1984, by a team from the pediatric dentistry division of the Karolinska Institute, headed by the Swedish researcher Barbo Malmgren.^2^ With the resources available at the time, the dentist had very little or no option to treat this condition, especially in patients who still had a lot of growth potential and who would suffer from the consequences of the ankylosis for an important period of their lives - childhood and adolescence.^4^

In the 80s, available treatment possibilities included to keep the patient’s tooth until the crown fell out, or proceed with its extraction.^2^ ^,^ ^11^ Malmgren’s study completely changed this paradigm, proposing a new method for managing dentoalveolar ankylosis.^2^

The decoronation procedures attempt to overcome the deleterious effects that an ankylosed teeth extraction could bring to the alveolar bone, as well as the esthetic damage that the tooth in infraposition could have if the patient/dentist chose not to perform any intervention until the crown had fallen off.^2^ In addition to the aesthetic issue, studies show that the more severe the level of infraposition, the more complex the future rehabilitation process.^2^ ^,^ ^5^ ^,^ ^10^

Even though this therapy is extremely well-supported by the Swedish investigators,^2^ ^,^ ^11^ today, almost 40 years after its first publication, a significant number of dental professionals and researches don’t advocate it, and a few studies are available in the literature. The more frequent updates have also been conducted by Malmgren’s own research team.^3^

This fact is corroborated by a systematic review focusing on the effectiveness of this technique^4^. It states that decoronation is poorly disseminated worldwide (reinforcing what is observed in private practices and orthodontic schools). The authors attribute it to the reduced number of publications demonstrating its rate of success. Outside commercial interests may be considered as an extrinsic variable influencing the sale of materials that supposedly have the potential to stop the ankylosis process.^4^ ^,^ ^12^

Also confirming the findings of this study, a group of researchers investigated the knowledge about decoronation interviewing 120 undergraduate dental students and 200 master’s dental students in Italy, and found that only 6.2% reported knowing the technique, and among these, only 1.5% were able to correctly describe the whole technic.^5^

Decoronation is based on physiological principles that refer to some concepts of basic embryology. It is important to remember that bone and dental tissues, when in direct contact, fuse spontaneously.^13^ However, in the evolution of the human species, two crucial factors came into play: the Malassez Epithelial Rests and the growth factors (EGF) released by this epithelium and present in the periodontal ligament.

The presence of bone cells, the EGF, in the periodontal ligament stimulates their resorption by osteoclasts, which then prevents contact with the dental tissue, not allowing the fusion of cement and bone.^13^

This scenario happens under conditions of perfect homeostasis; however, when severe trauma occurs, the necrosis of Malassez epithelial rests may be present, which leads to the lack of protection against undesired contact between bone and cementum, triggering the ankylosis process.^13^

It is necessary to differentiate this process from a pulp pathology triggered by a carious disease, which has an infectious character. Considering the traumatic pathology from which ankylosis results, it occurs in a septic condition, through a natural process, without the presence of infection and inflammatory exudate. Therefore, natural resorption is observed.^13^

As this is a physiological process, Malmgrem pictured the possibility of maintaining the roots until the organism itself completed its fusion in bone tissue, bringing a beneficial effect for the patient, eliminating the possibility of bone loss that would likely occur in an extraction, since it would be transformed (after 1 to 10 years) into complete bone tissue. It can be considered a natural bone graft in that area.^2^ ^,^ ^11^

Relying on the principles of immunology, the author consolidated the idea of ​​forming a blood clot inside the tooth, stimulating an inflammatory process towards the repair pathway (TH2), helping the process of fusion between bone and cementum.^2^

It is necessary to remove all the endodontic material present, since it could behave as an irritating and non-biological material, delaying the renewal of cementum into bone matrix.^2^ ^,^ ^11^ Reinforcing this finding, a publication prior to Malmgrem’s evaluated bone preservation in replanted and submerged dental roots of dogs, and showed that the presence of endodontic material altered the tissue repair process,^14^ while fusion into bone tissue was more pronounced in teeth with vital roots.^3^ ^,^ ^14^

Regarding the aesthetic issue created by the infraposition of teeth in growing individuals after ankylosis development, Malmgrem idealized the complete removal of the crown at the cementum-enamel junction and then proceed with an adhesive crown when growths end.^2^

The question of how rehabilitation occurs is poorly described in studies on decoronation.^10^ At the time of Malmgrem’s first article, few options were available;^2^ however, nowadays, there have been advances in restorative dentistry, such as the advent of implants. In this review, we found only a single systematic review focused on the rehabilitation of post-decoronation teeth, which states that the best way to rehabilitate is through a modified lingual/palatal arch with a prosthetic crown^10^. The same study cites the imperative need for such rehabilitation by a multidisciplinary team.^10^

### THE DESCRIPTION OF THE TECHNIQUE

The decoronation procedure remains faithful to what was proposed in the first study in 1984.^2^ ^,^ ^11^ It is a simple technique, in which a mucoperiosteal flap is opened initially in the area of the ankylosed tooth. The entire crown is removed at the level of the cementum-enamel junction using a diamond drill, followed by the removal of all filling material present inside the root canal, and an intense saline irrigation allows the root canal system to fill with blood. Afterward, the root surface is reduced to 2mm below the bone level and the flap is simply sutured.^11^

### MAIN INDICATIONS

Patients’ growth stage is crucial in treatment planning in all areas of dentistry and would not be any different for decoronation.^4^ ^,^ ^11^ The first point that needs to be evaluated to suggest or not the use of this technique is determining the growth stage of the patient, verifying if the patient has already ceased his/her growth potential or is still in the early skeletal maturation stage.^4^ A patient whose maturational indicators clearly show that growth has ceased may not be a good candidate to be treated with decoronation. At this stage, it may be wiser to monitor the development until the crown falls, which may take a long time,^2^ and then proceed with a well-planned rehabilitation.^4^

The scenario changes dramatically when trauma affects a growing patient. This is the biggest and most important indication for decoronation, as these patients have their maxillary alveolar process in full development, which will result in an ankylosed tooth in an inferior position in relation to the adjacent ones.^2^ ^,^ ^4^

This aesthetic damage has a significant influence on the perception of the affected individuals, by their peers, and can trigger relevant psychosocial repercussions in their lives. The infraposition can lead to a complete inversion in the smile esthetics.^15^ In 2014, Machado^16^ stated that the central incisors are the protagonists of the smile.

In addition, the infraposition also results in greater severity of the condition.^4^ ^,^ ^15^ The younger the patient, the greater his/her growth potential, and the more critical the infraposition. The level of infraposition has been associated with a greater difficulty in future prosthetic rehabilitation.^2^ ^,^ ^10^

### PARTICULARITIES OF DECORONATION IN GROWING PATIENTS

In 2013, Malmgrem and her collaborators mentioned that the ankylosed tooth can, indeed, be kept in the mouth without any intervention, as a space maintainer, but for a reasonable time. This time ends when the first signs of intrusion in relation to its adjacent teeth begin to appear. At the first sign of infraposition, decoronation must be performed.^11^

The articles reviewed in the present article show that the success rate of the procedure and the time to intervene are linked to where the patient is in the growth curve.^2^ ^,^ ^4^ ^,^ ^11^ There seems to be a consensus that the ideal treatment starts before or during the growth spurt.^4^

Variables such as sex and individual growth must be considered when indicating decoronation.^4^ Some studies demonstrate that chronological age and the pattern of facial growth also need to be taken into account.^4^ ^,^ ^11^

Children younger than 10 years old at the time of the trauma may develop a more severe and faster condition, while those aged 10-12 years may take a longer time to develop an infraposition. At older ages, the data are not homogeneous. This fact is in agreement with the greater vertical growth of the maxilla during the eruption of permanent incisors and the greater growth rate.^4^

The ideal age to perform decoronation varied between studies and between sexes. A retrospective analysis^3^ found an average of 14.6 years for boys and 13 years for girls. More investigations seem to be necessary, but definitely sex is an important variable to consider when deciding when to treat. This can be explained due to different growth spurt time in boys and girls, being earlier in girls, and later in boys.^4^

The individual growth rate must be taken into account, since the infraposition rate showed a positive relationship with the individual growth curve, which becomes an important variable, since not all patients will go through the growth peak at the same time, and it may be necessary to advance or postpone the decoronation.^4^

### BONE TISSUE MAINTENANCE

One of the main benefits suggested by Malmgrem when conceiving this method was the preservation of bone tissue, both in height and width.^2^ ^,^ ^11^ In a study evaluating 14 ankylosed teeth, the author concluded that decoronation preserved bone tissue.^2^ Later, with a larger sample of 103 ankylosed elements, Malmgrem observed not only the maintenance of bone tissue, but an increase in the volume, mainly in patients treated before or during pubertal growth.^3^ ^,^ ^11^ The article further emphasized that girls should be treated earlier than boys.^3^ ^,^ ^11^ In line with this finding, a systematic review showed tissue maintenance or a gain of 1 mm of coronal bone 2 to 3 years after decoronation.^3^

When individually analyzing the gains in height and width, the same review cites that there is no consensus in the literature. All 12 evaluated studies showed preservation in bone height; but, concerning width, some articles showed a slight reduction after decoronation.^4^ Considering all the above and after reviewing the biological bases, indications and technique, a case report treated with decoronation will be presented.

## CASE REPORT

**Figure 1:**
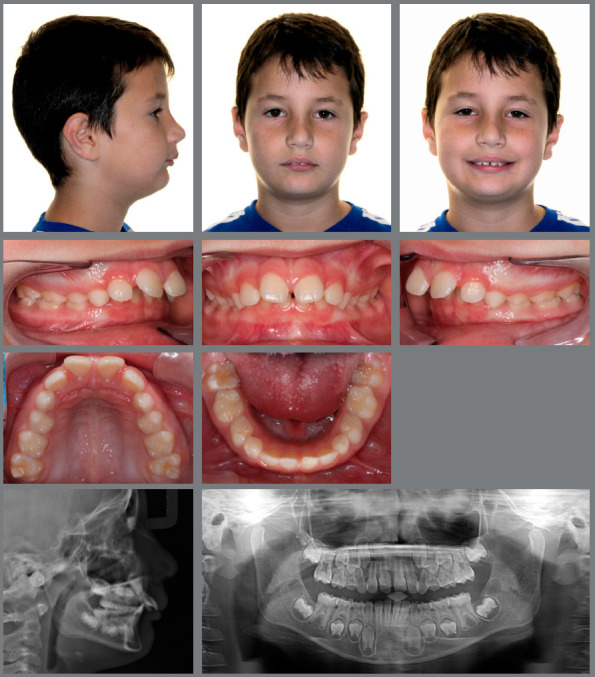
Pretreatment evaluation.

An 8 years and 3 months old male was under interceptive orthodontic treatment with the objectives of reducing his overjet, improving Class II relation, and reducing the overbite. Figure 1 shows the facial, dental and radiographic aspects of pretreatment. The treatment plan involved the use of cervical headgear and a 2x4 appliance.

**Figure 2:**
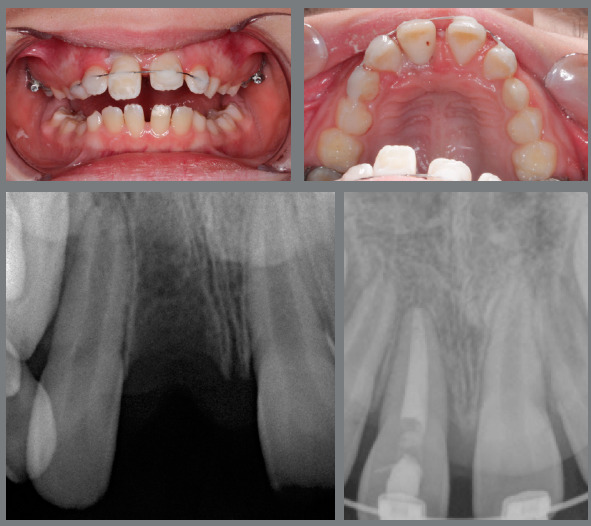
Clinical and radiographic aspects after reimplantation.

One year after the start of the orthodontic treatment, the patient suffered severe trauma, leading to avulsion of the maxillary right central incisor. Replantation and endodontic treatment were performed by another professional. He was followed-up at the endodontics department of Saint Louis University for four months (Fig 2). After a period of stabilization, the orthodontic treatment continued, with very light forces. The treatment plan was changed and, due to the trauma in the maxillary arch, the decision was to concentrate the efforts to correct the Class II on the mandibular arch with the help of a functional appliance. 

**Figure 3:**
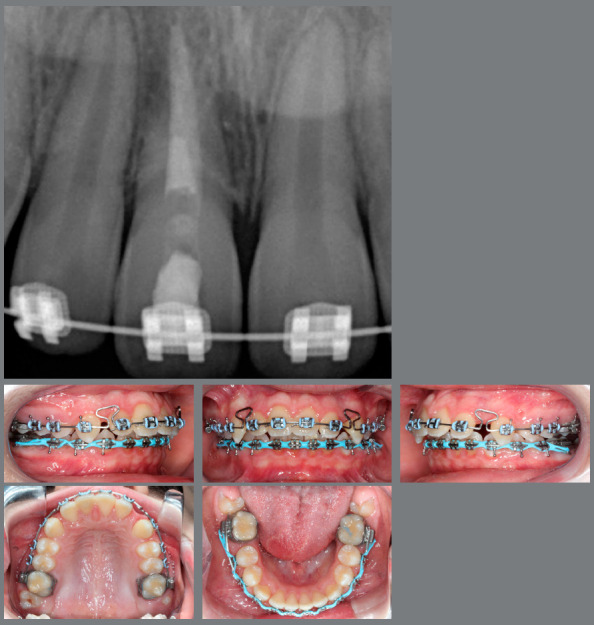
Radiograph and intraoral photographs showing the ankylosed tooth and the loss of anchorage during space closure.

Considering this fact, a Herbst appliance to correct the Class II was the recommended protocol. The results obtained with the functional appliance were satisfactory, with complete correction of Class II and some improvement of the facial pattern. An attempt to close the spaces in the maxillary arch was performed, but an adverse effect was noted. Instead of anterior retraction, a mesial movement of the entire posterior segment occurred. Such loss of anchorage, associated with the radiographic images, led to a final diagnosis that the tooth #11 was ankylosed (Fig 3), which imposed a new decision-making process.

**Figure 4:**
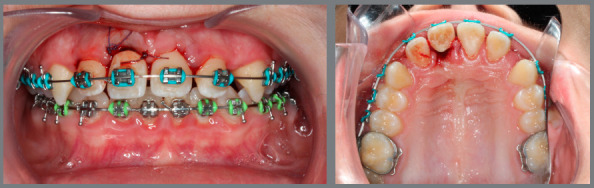
Immediate post-decoronation.

**Figure 5:**
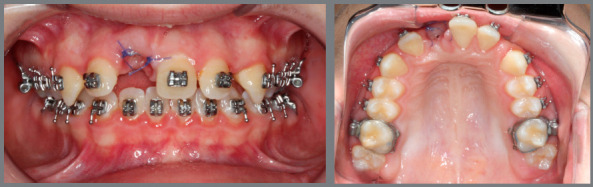
One week after decoronation.

Among the different possibilities to treat an ankylosed incisor, decoronation was the first choice for this patient, with the objective of preserving bone tissue in both height and width. To perform this, the crown of the tooth #11 was cut at the cementum-enamel junction, the root canal system was cleared, filled with abundant irrigation with saline solution and later with blood. The flap was then brought back into position, sutured, and then the patient’s own crown was used as a provisional tooth, attached to the orthodontic wire (Fig 4). Reinforcing the benefits to the periodontium already mentioned, the healing of periodontal tissue after just a week of the surgery confirmed the success of the procedure (Fig 5).

**Figure 6:**
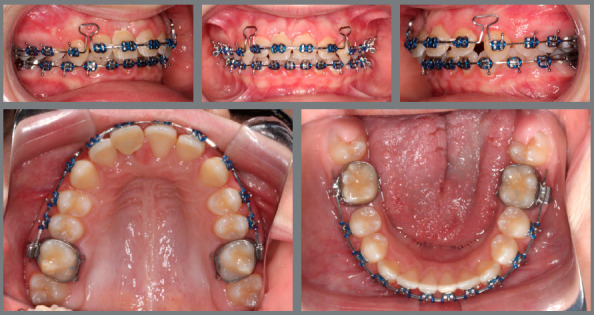
T-Loop for closing spaces.

The treatment proceeded by closing the spaces using a T-loop. Figure 6, two months after the decoronation, shows that the case followed the usual way and without any difficulties that ankylosis could bring to the case.

**Figure 7:**
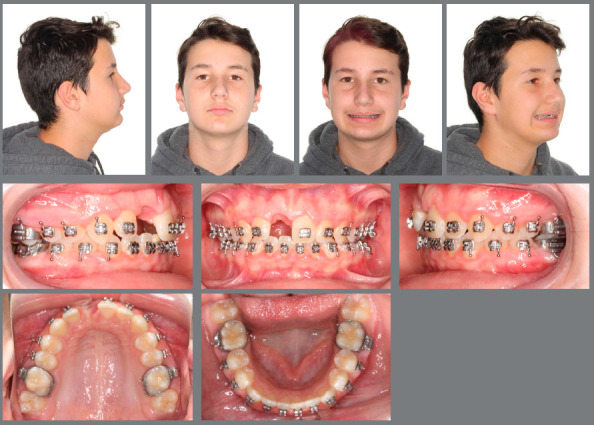
Dental aspects 6 months after decoronation.

Figure 7 shows adequate overjet and overbite, as well as an Angle Class I of molars and canines. The maxillary alveolar process was uniform and without any negative growth defect.

**Figure 8:**
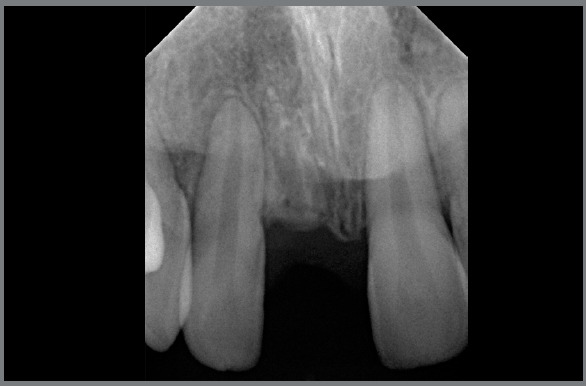
Radiographic aspects after eight months of decoronation.

In order to evaluate the bone level in width and height, as well as the process of fusion of the ankylosed root into bone, periapical radiographs were taken. Figure 8 shows the evolution of the process eight months after decoronation, in which can be observed that the root was in the process of remodeling into bone tissue, and that the volume was preserved, the main objective of decoronation.

### FINAL TREATMENT EVALUATION

**Figure 9:**
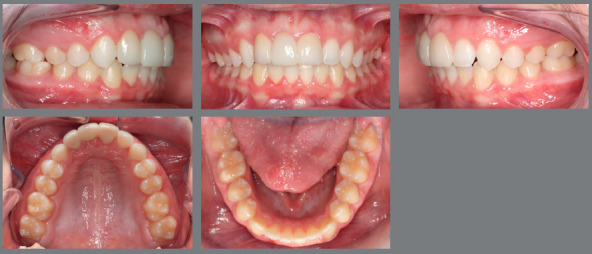
Final aspect of the treatment.

**Figure 10:**
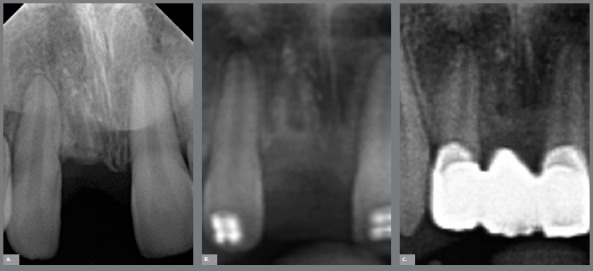
Decoronation follow-up: **A)** 8 months, **B)** 11 months, **C)** 19 months.

The treatment, which at first showed a small degree of complexity, had its perspective changed after the development of the ankylosis, to high severity, a situation that was overcome with the simple and effective use of the decoronation. Figure 9 shows the finished case, with good intercuspation, adequate molar and canine relation, and good overjet and overbite. The patient was later rehabilitated with a fixed adhesive bridge, a decision of the family and the general dentist. Normally, this is done according to a growth evaluation. Figure 10 shows a comparison of the cementum turnover into alveolar bone at three different time points.

## CONCLUSION

The present article demonstrates that ankylosis is an important sequelae of dental trauma, with high prevalence in the global population. It imposes itself as an interference and complication in orthodontics, especially in patients in active growth. Decoronation is recommended as an alternative for managing ankylosed teeth, but there are still few quality studies available in the literature.

 Professionals who are familiar with decoronation have in their arsenal of treatments a safe, effective, efficient and simple alternative for the treatment of this pathology, with preservation and maintenance of bone tissues, which allows orthodontic treatment to follow its usual course without major complications, allowing a result of excellence.
